# Endogenous expression of FAD-linked PS1 impairs proliferation, neuronal differentiation and survival of adult hippocampal progenitors

**DOI:** 10.1186/1750-1326-8-41

**Published:** 2013-10-20

**Authors:** Karthikeyan Veeraraghavalu, Se Hoon Choi, Xiaoqiong Zhang, Sangram S Sisodia

**Affiliations:** 1 Department of Neurobiology, The University of Chicago, 947 E 58th Street, AB 308, Chicago, Illinois 60637, USA

**Keywords:** Adult neurogenesis, Presenilin mutants, Alzheimer’s disease

## Abstract

**Background:**

Alzheimer’s disease (AD) is characterized by progressive memory loss and impaired cognitive function. Early-onset familial forms of the disease (FAD) are caused by inheritance of mutant genes encoding presenilin 1 (PS1) variants. We have demonstrated that prion promoter (PrP)-driven expression of human FAD-linked PS1 variants in mice leads to impairments in environmental enrichment (EE)-induced adult hippocampal neural progenitor cell (AHNPC) proliferation and neuronal differentiation, and have provided evidence that accessory cells in the hippocampal niche expressing PS1 variants may modulate AHNPC phenotypes, *in vivo*. While of significant interest, these latter studies relied on transgenic mice that express human PS1 variant transgenes ubiquitously and at high levels, and the consequences of wild type or mutant PS1 expressed under physiologically relevant levels on EE-mediated AHNPC phenotypes has not yet been tested.

**Results:**

To assess the impact of mutant PS1 on EE-induced AHNPC phenotypes when expressed under physiological levels, we exposed adult mice that constitutively express the *PSEN1 M146V* mutation driven by the endogenous *PSEN1* promoter (PS1 M146V “knock-in” (KI) mice) to standard or EE-housed conditions. We show that in comparison to wild type PS1 mice, AHNPCs in mice carrying homozygous (*PS1*^*M146V/M146V*^) or heterozygous (*PS1*^*M146V/+*^) *M146V* mutant alleles fail to exhibit EE-induced proliferation and commitment towards neurogenic lineages. More importantly, we report that the survival of newborn progenitors are diminished in PS1 M146V KI mice exposed to EE-conditions compared to respective EE wild type controls.

**Conclusions:**

Our findings reveal that expression at physiological levels achieved by a single *PS1 M146V* allele is sufficient to impair EE-induced AHNPC proliferation, survival and neuronal differentiation, *in vivo*. These results and our finding that microglia expressing a single *PS1 M146V* allele impairs the proliferation of wild type AHNPCs *in vitro* argue that expression of mutant PS1 in the AHNPC niche impairs AHNPCs phenotypes in a dominant, non-cell autonomous manner.

## Background

AD is pathologically characterized by the deposition of β-amyloid (Aβ) peptides in senile plaques and neuronal loss that leads to impairments in memory and cognitive function. Aβ peptides are derived from larger amyloid precursor protein (APP) by the concerted action of BACE1 and a multiprotein complex, termed γ-secretase, that catalyzes intramembranous proteolysis of a variety of type I membrane substrates. Inheritance of mutations in *PSEN1*, *PSEN2* or *APP* genes, encoding PS1, PS2 or APP variants, respectively, cause early-onset, autosomal dominant forms of familial AD (FAD) [1]. PS are the catalytic components of the γ-secretase complex and expression of PS1 variants leads to elevations in the ratio of Aβ42/Aβ40 peptides, leading to nucleation, oligomerization and neuropathogenicity of Aβ42 peptides [2]. Notwithstanding this important aspect of pathogenesis mediated by FAD-linked PS1 variants, a number of studies have revealed that PS1 influences multiple signaling pathways and have more global effects on neuronal function and plasticity [3].

Adult hippocampal neural stem cells (AHNSCs), a self-renewing cell population in the subgranular layer (SGL) of the dentate gyrus (DG), functionally mature into neurons and integrate into the hippocampal circuitry [4]. This process of adult neurogenesis has been implicated in hippocampal function, learning and memory, and adaptation to novel environments, stress response, depression, injury or disease [5-7]. Genetic ablation of new born neurons in adult mice results in gradual loss of granule cell numbers in the DG, leading to impairments in hippocampal-dependent behaviors, including contextual and spatial memory [8]. On the other hand, exposure of adult rodents to an enriched environment (EE), in which animals are placed in large cages containing running wheels, colorful tunnels and assorted toys, increases hippocampal neurogenesis and enhances spatial learning performance [9-11]. We have demonstrated that transgenic mice expressing FAD-linked PS1 variants in a ubiquitous manner exhibit impairments in EE-mediated proliferation and neuronal differentiation of AHNSCs [12], and that these AHNSC phenotypes are driven by non cell-autonomous mechanisms that require mutant PS1 expression in microglia [12] and excitatory neurons [13].

We now extend these latter observations and demonstrate that EE-induced AHNPC proliferation and neuronal differentiation is also impaired in PS1 M146V KI mice that harbor a germ line *M146V* mutation [14]. We also report that the conditioned medium of microglia obtained from PS1 M146V KI mice impairs the proliferation of wild type AHNPCs. Finally, we report that the survival of newborn progenitors are diminished in PS1 M146V KI mice exposed to EE-conditions compared to respective enriched wild type control cohorts.

## Results

### EE-induced AHNPC proliferation in mutant PS1 KI mice

**Figure 1:**
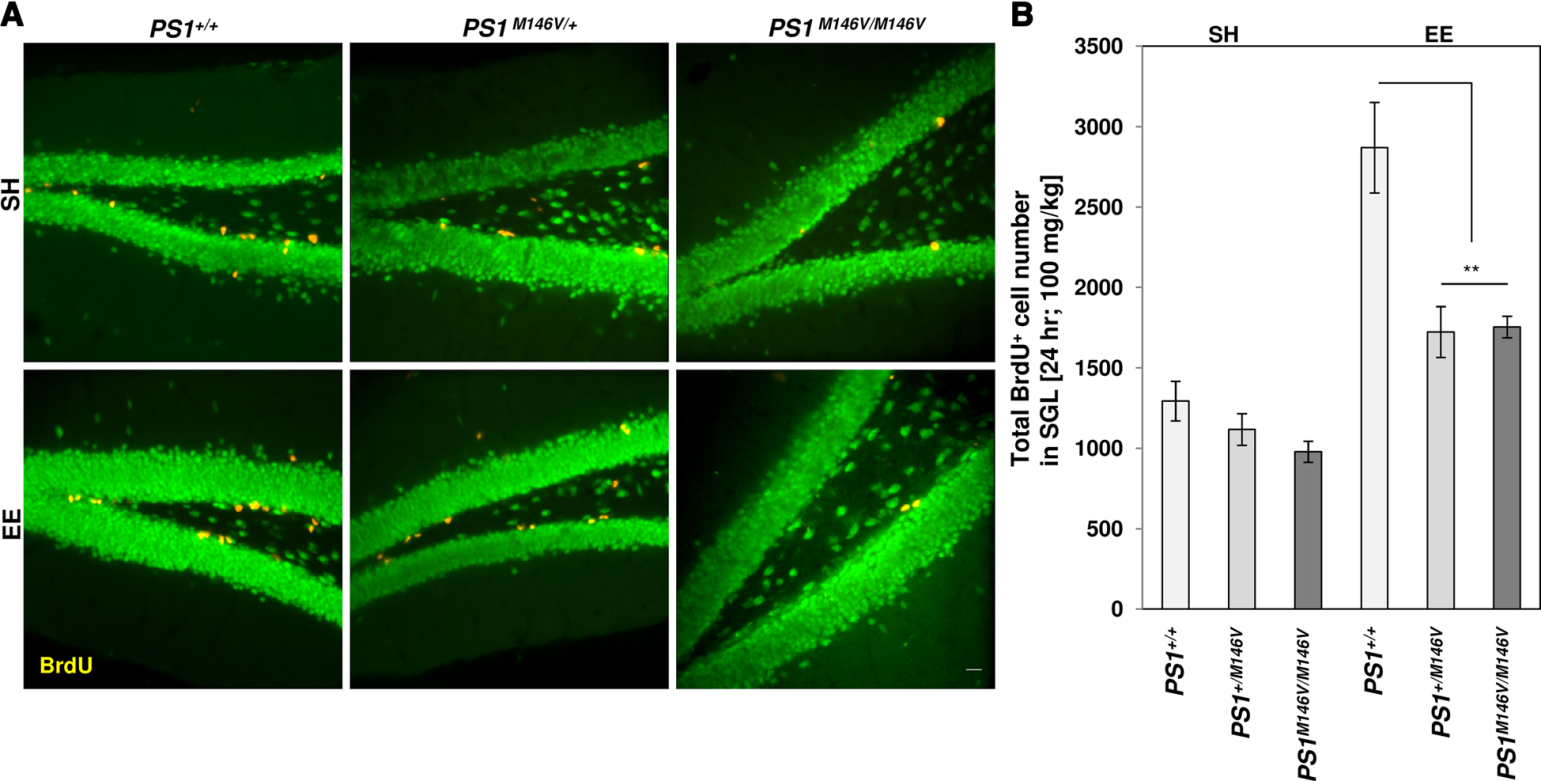
**AHNPC proliferation in PS1 M146V KI mice following exposure to SH or EE housing conditions. (A)** Representative images of BrdU+ AHNPCs (yellow) in the SGL of *PS1*^*+/+*^, *PS1*^*M146V/+*^ or *PS1*^*M146V/ M146V*^ mice, exposed to SH or EE conditions for one month, injected with a single i.p dose of 100 mg/kg of BrdU and sacrificed 24 hr later. NeuN, green. Scale bar, 100 μm. **(B)** Quantification of total BrdU+ cells in SGL. Two-way ANOVA followed by *post-hoc* Tukey test; *N* = 10, 10, 9 for SH animals, and 10, 12, 11 for EE animals (m ± SEM). ** *p* < 0.01.

A cohort of one month old male wild type (*PS1*^*+/+*^), heterozygous (*PS1*^*M146V/+*^) or homozygous (*PS1*^*M146V/M146V*^) mice were exposed to standard housing (SH) or EE conditions for one month. At the end of one month, animals housed under these two conditions were injected intraperitoneally (i.p) with a single dose of 100 mg/kg of BrdU. Mice were sacrificed 24 hr post injection, and brain sections were subjected to immunofluoresence analysis using a BrdU-specific antibody (Figure 1A), and the number of BrdU+ cells in SGL was quantified. Under SH conditions, no significant difference in BrdU+ cell numbers in the DG were observed between *PS1*^*+/+*^ and *PS1*^*M146V/+*^ mice (Figure 1B; *P*=0.644), or between *PS1*^*+/+*^, *PS1*^*M146V/M146V*^ mice (Figure 1B; *P*=0.266), or between *PS1*^*M146V/+*^ and *PS1*^*M146V/M146V*^ mice (Figure 1B; *P*=0.756). Exposure to EE lead to a significant increase in BrdU+ cell numbers in all three genotypes when compared to their respective control cohorts exposed to SH conditions (Figure 1B; *P*<0.01). However, and in contrast, with the a prominent ~2.2-fold increase in BrdU+ cell numbers that was detected in EE versus SH *PS1*^*+/+*^ mice, exposure to EE lead to only a modest 1.48- and 1.49-fold increase in BrdU+ cell numbers in *PS1*^*M146V/+*^ and *PS1*^*M146V/M146V*^ mice, respectively, compared with their SH cohorts. Moreover, the BrdU+ cell numbers in the hippocampus of *PS1*^*M146V/+*^ and *PS1*^*M146V/M146V*^ mice exposed to EE conditions were significantly lower than the numbers observed in enriched *PS1*^*+/+*^ mice (Figure 1B; *P*<0.01, EE *PS1*^*+/+*^ vs. EE *PS1*^*M146V/+*^ and EE *PS1*^*+/+*^ vs. EE *PS1*^*M146V/M146V*^).

### EE-mediated differentiation and survival of AHNCPs in mutant PS1 KI mice

**Figure 2:**
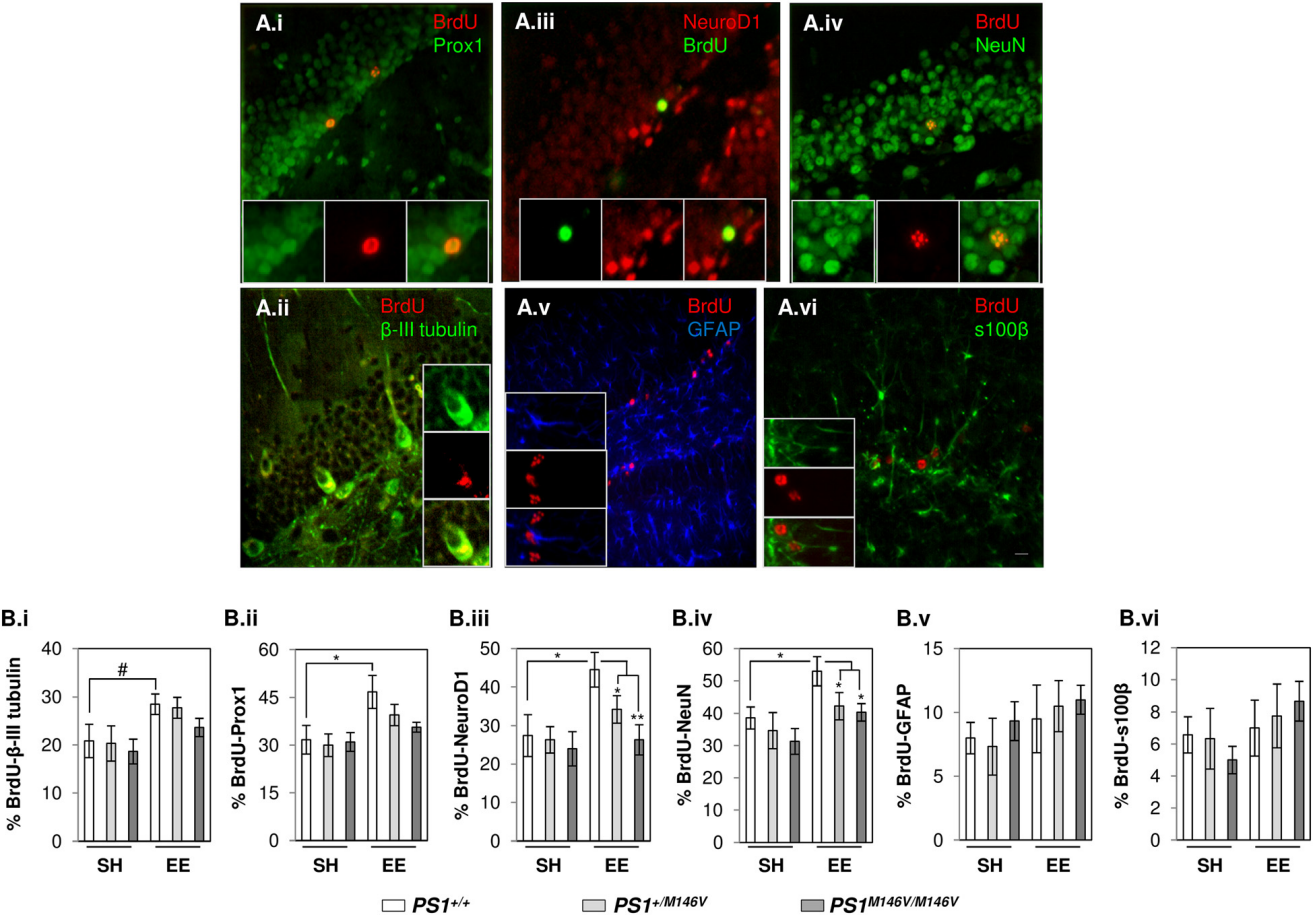
**EE-induced differentiation of AHNPC in PS1 M146V KI mice. (A)** Representative images of BrdU+/Prox1+ (A.i), BrdU+/β-III tubulin+ (A.ii), BrdU+/NeuroD1+ (A.iii), BrdU+/NeuN+ (A.iv), BrdU+/GFAP+ (A.v) or BrdU+/s100β+ (A.vi) co-labeled cells in *PS1*^*+/+*^ mice exposed to EE condition, injected with a single i.p dose of 100 mg/kg of BrdU and sacrificed 2 weeks later. Scale bar, 25 μm. Inserts inside each panel shows subfield of images captured through individual channels and the respective overlays highlighting cells co-labeling of BrdU and mentioned antigenic marker of differentiation. **(B)** Quantification of co-labeled cells with BrdU and markers of differentiation in DG of *PS1*^*+/+*^, *PS1*^*M146V/+*^ or *PS1*^*M146V/ M146V*^ mice exposed to SH or EE housing condition. Two-way ANOVA followed by *post-hoc* Tukey test for analyzing multiple groups or *T*-test; *N* = 7, 8 (SH vs. EE, *PS1*^*+/+*^); 6, 8 (SH vs. EE, *PS1*^*M146V/+*^); 6, 6 (SH vs. EE, *PS1*^*M146V/ M146V*^) (m ± SEM). * *p* < 0.05, ** *p* < 0.01, ^#^*p* = 0.075.

To assess the impact of the PS1 M146V KI mutation on EE-induced differentiation of newly-born AHNPC into neuronal or glial lineages, we exposed cohorts of *PS1*^*+/+*^, *PS1*^*M146V/+*^ or *PS1*^*M146V/M146V*^ mice to SH or EE conditions for one month, and then injected these animals with a single dose of BrdU (100 mg/kg). The cohorts of *PS1*^*+/+*^, *PS1*^*M146V/+*^ or *PS1*^*M146V/M146V*^ mice were then returned to respective SH or EE conditions for an additional 2 weeks. We chose the two week time point for analysis because this is an accepted time window to score for neuronal or glial lineage commitment of AHNPCs and their survival efficiency [15]. More specifically, this earlier time point allows us to score the proportion or percentage of AHNPCs that express different neuronal lineage markers, such as the early immature (β-III tubulin), early mature (Prox1), mature (NeuroD1) and terminally differentiated (NeuN) stages. At the end of two weeks, the animals were sacrificed and brain sections were subjected to immunofluorescence staining with anti-BrdU antibodies combined either with antibodies against: TuJ1 (βIII tubulin), a marker of both early immature and mature neurons; Prospero-related homeobox 1 (Prox1), a marker of early and late mature neuronal lineages; NeuroD1, a marker for mature neuronal lineages; NeuN, a marker for mature neurons; or glial fibrillary acidic protein (GFAP) and s100β, markers for astrocytes. Representative images are shown in Figure 2A.

Quantification of BrdU+ cells co-labeled with lineage specific markers showed no significant differences across all the genotypes under SH conditions (Figure 2B.i-vi). Exposure of *PS1*^*+/+*^ mice to EE resulted in a 1.366-fold increase, albeit insignificant, in the percentage of BrdU+/βIII tubulin+ cells compared with the SH cohort (Figure 2B.i; *P*=0.075; SH *PS1*^*+/+*^ vs. EE *PS1*^*+/+*^). Similarly, in comparison with the cohorts maintained in SH conditions, exposure of *PS1*^*M146V/+*^ or *PS1*^*M146V/M146V*^ mice to EE conditions resulted in an increase in the percentage of BrdU+/βIII tubulin+ cells by 1.364- and 1.267-fold, respectively (Figure 2B.i; *P*=0.091, SH *PS1*^*M146V/+*^ vs. EE *PS1*^*M146V/+*^; *P*=0.0147, SH *PS1*^*M146V/M146V*^ vs. EE *PS1*^*M146V/M146V*^).

Exposure of *PS1*^*+/+*^ mice to EE resulted in a significant, 1.474-fold increase in the percentage of BrdU+/Prox1+ cells compared to SH cohorts (Figure 2B.ii; *P*=0.04; SH *PS1*^*+/+*^ vs. EE *PS1*^*+/+*^). In contrast, in comparison with the cohorts maintained in SH conditions, exposure of *PS1*^*M146V/+*^ or *PS1*^*M146V/M146V*^ mice to EE conditions resulted in resulted in an insignificant 1.316- and 1.15-fold increase in the percentage of BrdU+/Prox1+ cells, respectively (Figure 2B.ii; *P*=0.078, SH *PS1*^*M146V/+*^ vs. EE *PS1*^*M146V/+*^; *P*=0.299, SH *PS1*^*M146V/M146V*^ vs. EE *PS1*^*M146V/M146V*^).

Analysis of cells colabeled with BrdU and the late neuronal markers, NeuroD1 and NeuN revealed that exposure of *PS1*^*+/+*^ mice to EE resulted in a significant increase by 1.62- and 1.37-fold in the percentage of BrdU+/NeuroD1+ or BrdU+/NeuN+ double labeled cells, compared with SH cohorts, respectively (Figure 2B.iii-iv; *P*=0.012 BrdU+/NeuroD1+ and 0.027 BrdU+/NeuN+; SH *PS1*^*+/+*^ vs. EE *PS1*^*+/+*^). On the other hand, exposure of *PS1*^*M146V/+*^ and *PS1*^*M146V/M146V*^ mice to EE conditions resulted in a insignificant increase in the percentage of BrdU+/NeuroD1+ or BrdU+/NeuN+ double labeled cells (Figure 2B.iii-iv; *P*=0.146, SH *PS1*^*M146V/+*^ vs. EE *PS1*^*M146V/+*^ and *P*=0.703, SH *PS1*^*M146V/M146V*^ vs. EE *PS1*^*M146V/M146V*^ for BrdU+/NeuroD1+; *P*=0.291, SH *PS1*^*M146V/+*^ vs. EE *PS1*^*M146V/+*^ and *P*=0.091, SH *PS1*^*M146V/M146V*^ vs. EE *PS1*^*M146V/M146V*^ for BrdU+/NeuN+).

Importantly, a significant reduction in the percentage of BrdU+/NeuroD1+ and BrdU+/NeuN+ double labeled cells was observed between *PS1*^*+/+*^ versus *PS1*^*M146V/+*^ or *PS1*^*M146V/M146V*^ groups that were exposed to EE conditions (Figure 2B.iii-iv; *P*=0.03 for BrdU+/NeuroD1+ and *P*=0.045 for BrdU+/NeuN+ in EE *PS1*^*+/+*^ vs. EE *PS1*^*M146V/+*^ mice; and *P*=0.006 for BrdU+/NeuroD1+ and *P*=0.024 for BrdU+/NeuN+ in EE *PS1*^*+/+*^ vs. EE *PS1*^*M146V/M146V*^ mice). Examination of glial lineages revealed no significant changes in the percentage of BrdU+/GFAP+ or BrdU+/s100β+ double-labeled cells between SH versus EE groups or within EE groups, across all three genotypes (Figure 2B.v-vi).

**Figure 3:**
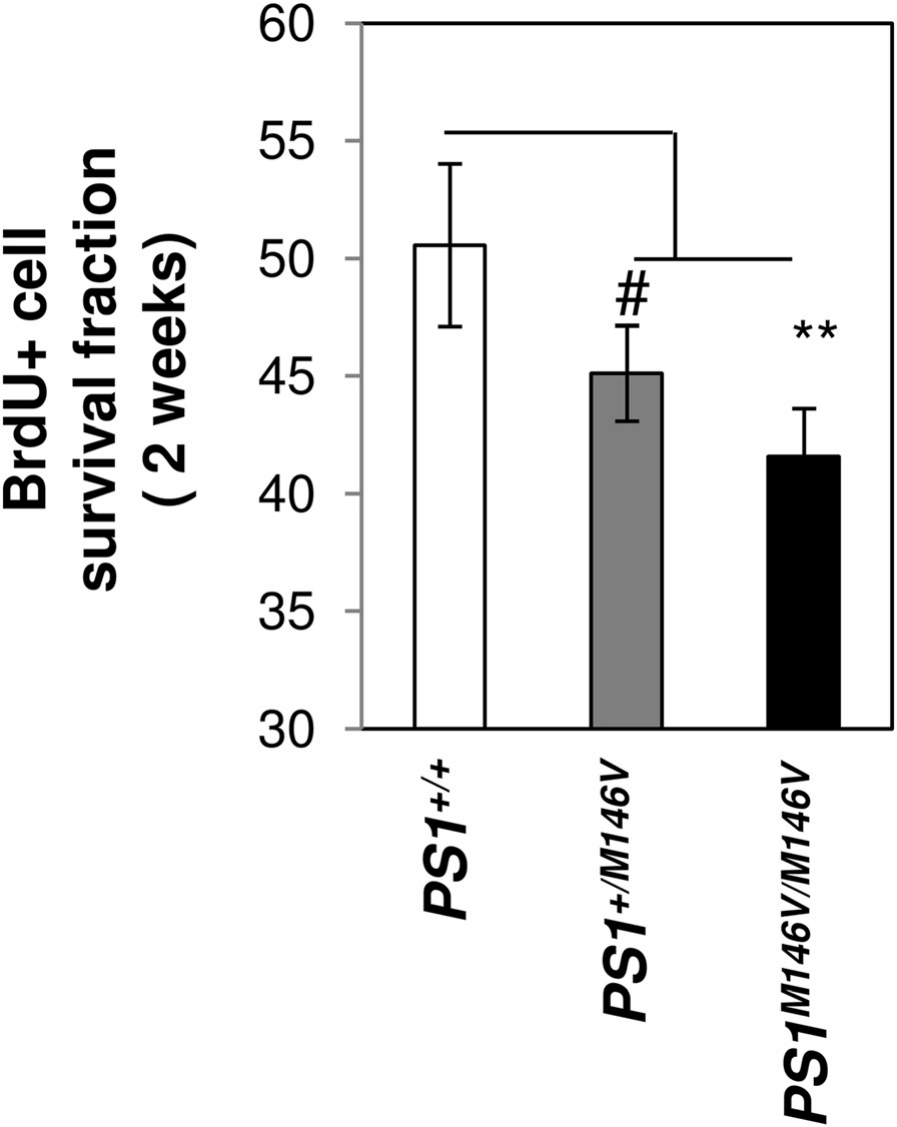
**Survival of AHNPC from PS1 M146V KI mice following exposure to EE housing conditions.** Histogram shows the survival fraction of BrdU+ AHNPCs in SGL and granular cell layers of PS1 M146V KI following exposure to EE housing conditions. Survival fraction, calculated as a percentage of the BrdU+ cell numbers remaining after 2 weeks over the numbers obtained when animals were sacrificed 24 hr after single dose of 100 mg/kg of BrdU. ANOVA followed by *post-hoc* LSD test; *N* = 8, 8, 6 for EE *PS1*^*+/+*^, *PS1*^*M146V/+*^ and *PS1*^*M146V/ M146V*^ mice, respectively. ** *p* < 0.01; ^#^*p* = 0.051.

To determine whether the observed impairments in neuronal differentiation of AHNPCs in mutant PS1 KI mice (Figure 2), might be a reflection of decreased survival of new born progenitors under EE conditions, we calculated the fraction of surviving BrdU+ cells in the SGL and granule cell layers as a percentage of the BrdU+ cell numbers remaining after 2 weeks compared to the numbers obtained when animals were sacrificed 24 hr after single dose of 100 mg/kg of BrdU (Figure 1B). While 50.56±2.17% of new born, BrdU-labeled AHNPCs survived for 2 weeks in *PS1*^*+/+*^ mice, a lower percentage of only 45.11±1.76% and 41.59±1.67% survived in *PS1*^*M146V/+*^ and *PS1*^*M146V/M146V*^ mice, respectively (Figure 3). One-way ANOVA followed by post-hoc LSD test revealed a near-significant *P* value of 0.051 in comparisons of *PS1*^*+/+*^ vs. *PS1*^*M146V/+*^ mice and a significant *P* value of 0.005 in comparisons of *PS1*^*+/+*^ vs. *PS1*^*M146V/M146V*^ mice. Collectively, these observations lead us to conclude that endogenous *PSEN1* promoter-driven expression of mutant PS1 decreases the survival of new born AHNPCs following EE.

### Conditioned medium (CM) from microglia expressing the PS1 M146V KI variant impairs proliferation of AHNPCs *in vitro*

**Figure 4:**
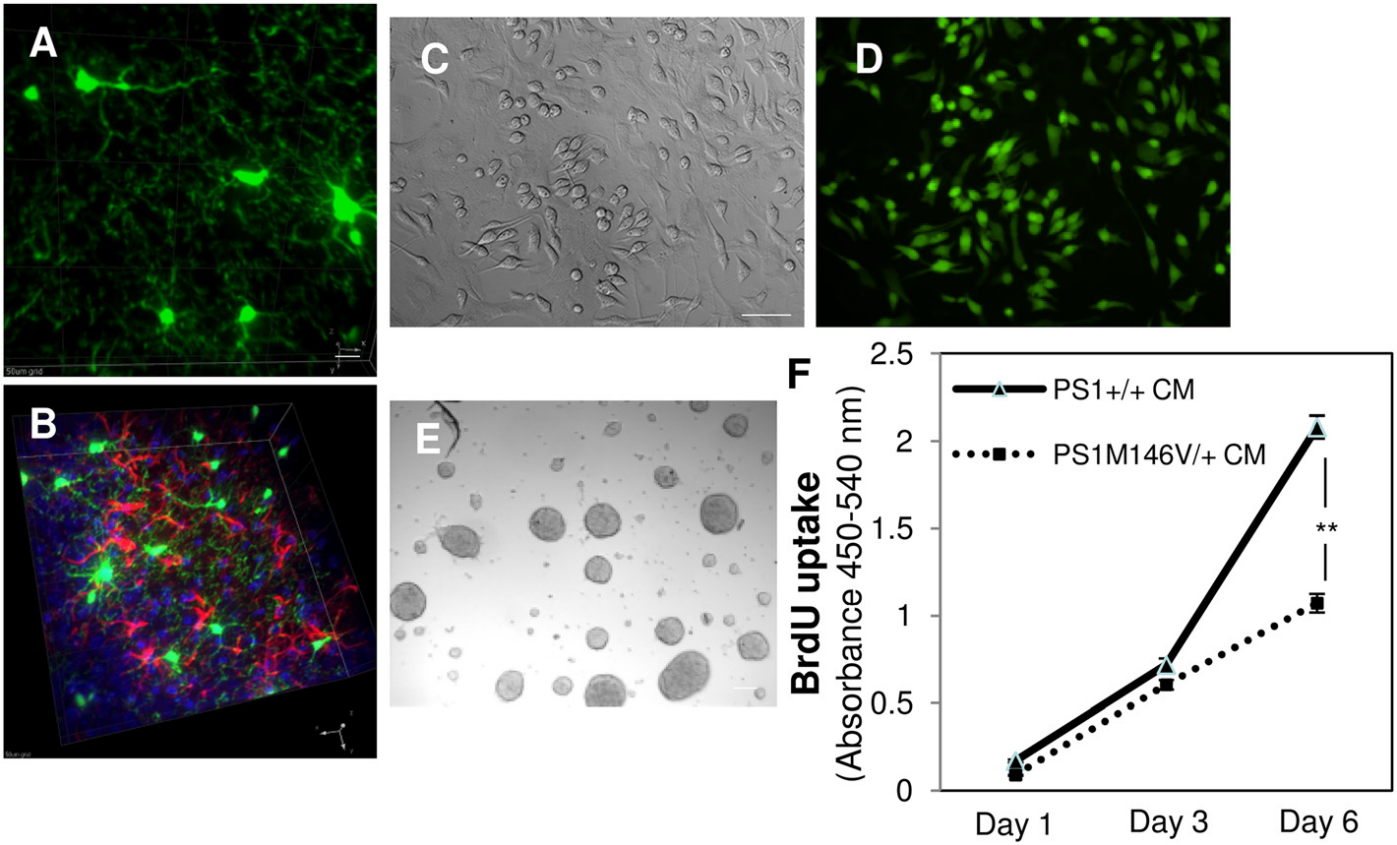
**Microglia conditioned media from PS1 M146V KI mice impairs proliferation of AHNPCs.** Representative image eGFP+ microglia (green) with ramified process in DG of *PS1*^*M146V/+*^*x Cx3Cr1*^*GFP/+*^ mice **(A)** and are negative for astrocytic marker, GFAP (red) **(B)**. Bright field **(C)** and fluorescent **(D)** images of flow cytometry sorted primary eGFP+ cultures established from neonatal brains of *PS1*^*M146V/+*^*x Cx3Cr1*^*GFP/+*^ mice. Scale bar, 25 μm. Bright field image of AHNPCs grown as neurospheres from *PS1*^*+/+*^, mice **(E)**. Scale bar, 50 μm. **(F)** Proliferation achieved by *PS1*^*+/+*^ AHNPCs following treatment with CM collected from IL-4 activated or *PS1*^*+/+*^*x Cx3Cr1*^*GFP/+*^*or PS1*^*M146V/+*^*x Cx3Cr1*^*GFP/+*^ microglia. Graph shows the extent of BrdU uptake in AHNPCs observed on days 1, 3 and 6. *T*-test; *N* = 3 independent cultures, with quadruplicates for each time point (m ± SEM). ** *p* < 0.01.

In earlier studies, we reported that both proliferation and neuronal cell fate commitment of AHNPCs from wild type mice was impaired by the addition of CM from IL-4-treated microglia obtained from transgenic mice that express PrP-driven FAD-linked PS1 mutants [12]. To test whether the CM from microglia obtained from *PS1*^*M146V/+*^ mice exhibit similar properties on AHNPCs, we bred either *PS1*^*+/+*^ or *PS1*^*M146V/M146V*^ mice to *Cx3Cr1*^*GFP/GFP*^ reporter mice in which cDNA encoding enhanced GFP (eGFP) has been knocked-in downstream of the fractalkine receptor *Cx3Cr1* promoter [16], leading to eGFP expression exclusively in CNS microglia with ramified processes (Figure 4A), but not in GFAP-expressing astrocytes (Figure 4B). Mixed glial cultures from neonatal *PS1*^*+/+*^*x Cx3Cr1*^*GFP/+*^ and *PS1*^*M146V/+*^*x Cx3Cr1*^*GFP/+*^ lines were established and eGFP+ microglia fraction was isolated by flow cytometry to establish pure cultures of microglia (Figure 4C and D). The GFP+ microglia established from *PS1*^*+/+*^*x Cx3Cr1*^*GFP/+*^ and *PS1*^*M146V/+*^*x Cx3Cr1*^*GFP/+*^ mice were activated with IL4 and the CM was incubated with AHNPCs from *PS1*^*+/+*^ mice that were cultured as neurospheres (Figure 4E). The proliferative capacity of AHNPCs was determined using a BrdU-uptake assay, as described earlier [12]. We observed that the extent of BrdU incorporation was similar across the AHNPCs cultures treated with microglia CM from *PS1*^*+/+*^*x Cx3Cr1*^*GFP/+*^ and *PS1*^*M146V/+*^*x Cx3Cr1*^*GFP/+*^ mice after 1 or 3 days, but by day 6, the proliferation of AHNPC was significantly reduced in the presence of CM from *PS1*^*M146V/+*^*x Cx3Cr1*^*GFP/+*^ microglia (Figure 4F; *P*<0.01). These results reveal that the impairments in proliferation of cultured AHNPCs observed earlier with CM from transgenic mice overexpressing FAD-linked *PSEN1* variants [12], can be recapitulated when an FAD-linked PS1 mutation is expressed at physiological levels when driven by the endogenous *PSEN1* promoter.

## Discussion

Earlier studies have shown that hippocampal neurogenesis declines with age and this reduction has been linked with impaired learning, memory, and deterioration of cognitive ability in the elderly [17-19]. In this regard, a recent report revealed that hippocampal neurogenesis is markedly reduced in patients with AD [20], findings which suggest that reductions in the number of new born granule cell neurons might enhance the rate of decline in hippocampal function and cognitive ability, and thereby significantly contribute to AD type dementia. In order to assess the potential role of FAD-linked PS1 variants on adult hippocampal neurogenesis, we and others have examined transgenic mice expressing PS1 variants driven by heterologous promoters [12,21-23]. However, a consensus regarding the role of mutant PS1 on AHNPC phenotypes has not emerged, and likely reflects the impact of overexpressing the transgenes in restricted cellular populations. For example, expression of the FAD-linked PS1P117L mutant driven by the neuron-specific enolase promoter failed to promote neurogenesis under SH conditions [23], and impaired survival of AHNPCs and neuronal differentiation under EE conditions [22]. However, expression of FAD-linked PS1A246E mutant driven by the neuron-specific Thy-1 promoter on a *PS1*^*-/-*^ background resulted in an increase in proliferation and decrease in survival of AHNPCs with no apparent reduction in net neuronal differentiation in animals exposed to SH conditions [21]. On the other hand, we reported that transgenic mice expressing the FAD-linked PS1ΔE9 and PS1M146L variants driven by the ubiquitously active PrP-promoter, fail to exhibit EE-induced AHNPC proliferation or differentiation towards neuronal lineage, with no noticeable changes in these parameters in SH conditions compared to mice expressing hPS1WT [12]. In this regard, PS1 has been shown to be expressed endogenously in AHNPCs [24], neurons [25], cerebral vasculature [26], glia and oligodendrocytes [12,27]. Thus, while it seems reasonable that ubiquitous, PrP promoter-driven of mutant *PSEN1* transgenes reflects the expression patterns of endogenous *PSEN1*, there remain concerns regarding expression levels and regulation in specific CNS cell types.

In order to address these potential concerns, we asked whether the EE-mediated impairments on AHNPC proliferation and neuronal differentiation that we documented in transgenic mice expressing FAD-linked PS1could be validated in “knock-in” mice harboring an FAD-linked PS1 mutation in the endogenous *PSEN1* locus, and we now offer several important insights.

First, we show that EE-induced proliferation of AHNPCs is impaired in both *PS1*^*M146V/+*^ and *PS1*^*M146V/M146V*^ animals, similar to our earlier findings in PrP-driven FAD-linked mutant *PSEN1* transgenic mice [12]. Interestingly, under SH conditions, we also observed a moderate reduction in proliferation of AHNPCs in *PS1*^*M146V/+*^ and *PS1*^*M146V/M146V*^ mice compared with wild type animals.

Second, we failed to detect significant changes in early immature and late neuronal lineages (BrdU+/βIII tubulin or BrdU+/Prox1, respectively) or glial lineages following EE. Instead, we observed a prominent reduction in the percentage of new born progenitors that differentiated towards mature neuronal NeuN+ and NeuroD1+ lineages in *PS1*^*M146V/+*^ and *PS1*^*M146V/M146V*^ mice. The lack of obvious changes in immature neuronal lineages correlates with the observations reported earlier in FAD-linked *PSEN1P264L* KI mice [28]. Consistent with the reduction in the number of new born neurons generated under EE conditions, the numbers of BrdU+ cells that survive two weeks post labeling were also diminished in *PS1*^*M146V/+*^ and *PS1*^*M146V/M146V*^ mice. It should be noted that we chose a two week time point for these latter analysis in order to score neuronal or glial lineage commitment of AHNPCs and their survival efficiency. The limitation of this analysis is that cells expressing mature neuronal markers may not be functionally integrated into the hippocampal network.

It is presently unclear whether the reduction in AHNPC proliferation and mature neuronal differentiation in *PS1*^*M146V/+*^ and *PS1*^*M146V/M146V*^ mice are a reflection of reductions in PS1 “activity”, but earlier studies by Wang and colleagues offer an important insight [29]. In the latter studies, AHNPC proliferation in standard housed *PS1*^*M146V/-*^ mice, wherein the mutant allele was placed in a *PSEN1* KO background, exhibited significant reductions in both proliferation and neurogenic potential of AHNPC compared with *PS1*^*M146V/+*^ mice [29]. The authors concluded that wild-type PS1 may play a “protective” role over the effects of the mutant M146V variant. While the nature of the PS1-dependent “activity” remains to be ascertained, our demonstration that lowering the levels of wild-type PS1 leads to reduced AHNPC proliferation and neuronal differentiation studies, taken together with the findings reported by Wang and colleagues [29], would be consistent with the idea that mutant PS1 exhibits a partial “loss of function”, as has been proposed earlier [30].

Third, we report that the survival of BrdU-labeled AHNPCs from *PS1*^*M146V/+*^ and *PS1*^*M146V/M146V*^ mice exposed to EE were significantly reduced compared with *PS1*^*+/+*^mice. While the molecular mechanism(s) underlying this aspect of mutant PS1 function is presently unclear, evidence from several earlier studies have accrued that support our observations [31-33]. For example, Guo et al. [14] reported that hippocampal neurons in *PS1*^*M146V/M146V*^ mice exhibit enhanced susceptibility to kianate-induced necrosis and that primary neurons from these mice showed enhanced sensitivity to glutamate-induced excitotoxicity. These results in *PS1*^*M146V/M146V*^ mice were confirmed independently in PrP promoter-driven transgenic mice expressing the L286V variant [31]. At a mechanistic level, it has been reported that reported that *PS1*^*M146V/M146V*^ mice exhibit disrupted intracellular Ca_2_+ signaling in neurons [33], suggesting that neurons expressing mutant PS1 variants have a lower threshold for excitotoxicity-mediated degeneration (reviewed in [34]). In this regard, we have demonstrated enhanced vulnerability of excitatory neurons in layer 2 (ECL2) of the entorhinal cortex in transgenic mice expressing the FAD-linked PS1ΔE9 variant following perforant pathway (PP) transection, compared with PP-lesioned transgenic mice expressing wild-type human PS1 [35].

Finally, we report that CM of microglia from *PS1*^*M146V/+*^ mice inhibits proliferation of AHNPCs derived from *PS1*^*+/+*^mice. These findings support our earlier observations using CM of microglia from mice expressing PrP-driven *PSEN1* transgenes [12]. The identity of the factors in mutant microglia CM and signaling pathways that are responsible for suppression of AHNPC proliferation are not fully understood. However, we showed earlier that eotaxin (or CCL11), Cxcl16, leptin and TIMP-1 (Tissue Inhibitor of Metalloprotease) are factors that were consistently elevated in the CM from microglia expressing FAD-linked PS1ΔE9 and PS1M146L variants [12]. In this regard, Wyss-Coray and colleagues [36] have offered a tantalizing insight. It is well-established that the proliferation and differentiation of AHNPC declines precipitously as a function of age [10], and Villeda et al. [36], sought to identify blood-borne factors in the systemic milieu that inhibit or promote adult neurogenesis in an age-dependent fashion in mice. One factor that was significantly elevated in the plasma of old mice was CCL11 that binds to, and activates the CCR3 chemokine receptor that is expressed by AHNPCs [37]. The nature of signaling pathways in AHNPCs that are activated upon binding of CCL11 or other factors secreted by microglia remain to be determined, and are areas of active investigation.

## Conclusion

We have documented that the EE-mediated impairments of proliferation, differentiation and survival of AHNPCs in mice with a knock-in of an FAD-linked *PSEN1* mutation fully recapitulates the findings observed in transgenic mice expressing PrP-driven FAD-linked *PSEN1* transgenes. These findings lead us to conclude that the memory deficits and cognitive decline in patients harboring *PSEN1* variants is a reflection not just of an increase in the ratio of Aβ42 to Aβ40, but to impairments in the self-renewal, survival and differentiation of AHNPCs.

## Methods

### Animals

*PS1*^*+/+*^, *PS1*^*M146V/M146V*^ KI mice [14], and *Cx3Cr1*^*GFP/GFP*^[16] were maintained in (C3H/HeJ x C57BL/6J F3) x C57BL/6J n1 background. *PS1*^*+/+*^ were crossed to *PS1*^*M146V/M146V*^ KI mice to obtain *PS1*^*M146V/+*^ line. The data are reported as mean ± standard error of mean (m ± SEM). Two-way ANOVA test (SPSS ver. 12), were performed for comparisons of quantitative data while analyzing data from multiple groups on AHNPC proliferation or differentiation studies. Tuckey or LSD test were used as a *post-hoc* analysis. *T*-test (Unpaired) was performed while comparing data from two independent data sets. Values of *p* < 0.05 were used as the criterion for statistical significance. Animal experiments were conducted in accordance with institutional and National Institutes of Health guidelines.

### EE setting and BrdU injections

For exposure to EE, cohorts of mentioned 1 month old male transgenic, wild type or PS1 M146V KI (heterozygous or homozygous) animals were housed in large cages containing running wheels, tunnels, toys, and chewable materials for 3 h a day for 1 month. Control groups of animals were maintained in standard laboratory housing conditions. Mice received a single i.p. injection of BrdU (100 mg/kg, Sigma, St. Louis, MO) on the last day of the enrichment. Half of the mice in each group were sacrificed 1 day after the injection to determine progenitor proliferation as described earlier [12]. For AHNPC differentiation studies, mice were allowed to continue under standard or EE conditions for 2 weeks and processed to determine survival and neuronal differentiation of the newborn cells.

### Tissue processing, estimation of proliferation and differentiation of AHNPCs

Tissue preparation, and immunofluorescent labelling for BrdU, neuronal nuclei (NeuN), β-III tubulin, Prox1, NeuroD1, s100β protein and Glial Fibrillary Acidic Protein (GFAP) were performed as described previously [12]. The antibodies used were rat anti-BrdU (1:100, Accurate Chemical & Scientific Corporation, Westbury, NY), mouse anti-NeuN (1:500, Chemicon, Temecula, CA), and rabbit anti-GFAP (1:500, Dako, Fort Collins, CO). The fluorescent secondary antibodies used were biotinylated donkey anti-rat IgG; Cy2-conjugated Streptavidin; donkey anti-mouse IgG conjugated with Cy5; donkey anti-rabbit IgG conjugated with Cy3 (all 1:250, Jackson ImmunResearch, West Grove, PA).

### Tissue preparation and immunostaining procedures

45 μM coronal sections of the brain were cut on a dry-ice cooled sliding microtome block (Leica, Wtzlar, Germany), serial sections spanning DG were collected in two rows of 24-well dish and stored at -20°C in a cryoprotective buffer and free floating sections were processed for immunohistochemistry or immunofluoresence as described previously [12], using the following primary antibodies: α-NeuN monoclonal antibody (1:800; Millipore, Temecula, CA), α-Prox1 polyclonal antibody (1:300; Millipore, Temecula, CA), α-BrdU rat monoclonal antibody (1:400; Accurate Chemical, Westbury, NY), α-βIII Tubulin TUJ1 monoclonal antibody (1:500; Covance, Emeryville, CA), α-GFAP polyclonal antibody (1:500; Dako, Fort Collins, CO) or α-s100β rabbit polyclonal antibody (1:500; Abcam, Cambridge, MA). To obtain total BrdU+ cell counts or co-labeling of BrdU with lineage specific antigens, every sixth and twelfth well sections from the 24-well plate, spanning the DG were stained with α-BrdU antibodies, and visualized using DyLight-549 conjugated donkey α-rat or α-rabbit antibody, respectively (1:400; Jackson ImmunoResearch, West Grove, PA). DNA denaturation, neutralization, blocking and washing steps for BrdU staining were followed as described earlier [12]. For double-labeling, a combination of DyLight-488, 549 or 649 conjugated secondary antibodies were used. Brain sections were visualized and imaged using an Olympus DSU Spinning disk inverted confocal microscope supported with EM-CCD Hamamatsu camera (Olympus Optical, Tokyo, Japan). Images at each wavelength were collected separately (DyLight-549, 555/617 nm), using a separate and specific excitation filter under x10 (0.4 NA, water), x20 (0.7 NA, water) or x40 (1.15 NA, water) objectives. Image acquisition settings were equivalent for all specimens, and were taken and recorded using Slide Book Software (Intelligent Imaging Innovations, Denver, CO). For estimating total BrdU+ or total double labeled cells with lineage specific markers, series of systematically selected every sixth section were stained and cells were counted throughout the coronal section in extent of the DG, by collecting images under x40 (1.15 NA, water) objective, equipped with Optronics Microfire CCD camera (Optronics, Goleta, CA) or imported from Slide Book library using DSU Spinning disk inverted confocal microscope. Cells were excluded from being counted when they intersected the top focal plane of the section as per the modified stereological procedure [38-40], and the sum of the counts was multiplied by six to obtain an estimate on total numbers. For co-labeling analysis of differentiated BrdU+ cell type, phenotype of 50 BrdU+ cells per animal were determined [12].

### AHNPC cultures, primary microglial cultures and proliferation assays

AHNPCs that give rise to neurospheres were cultured from dissected hippocampal tissue of 8 weeks old male *PS1*^*+/+*^ mice as described, previously [12]. In brief, single cell suspension from hippocampal tissue (~ 4 × 10^5^ viable cells/animal), were seeded in serum free culture media at a cell density of 50 cells/μl (SFM; Neurobasal medium containing 100 U/ml penicillin/streptomycin, 2 mM L-glutamine, 10 μg/ml of heparin, 20 ng/ml of b-FGF, 100 ng/ml of EGF and 2% B-27 supplement (Invitrogen, Carlsbad, CA)). Cultures were maintained at 37°C in 5% CO_2_/balance air. Proliferating neurospheres started to appear in about 10–12 days. To obtain single cell suspensions, the neurospheres were dissociated by trituration using Neurocult chemical dissociation solution (Stem cell tech, Toronto, Canada) and cultured through multiple passages. Primary cultures of mixed glia cells were prepared from hippocampus of newborn *PS1*^*+/+*^ or *PS1*^*M146V/+*^ mice according to previously described procedure [41]. In brief, hippocampal tissue from 8 to 16 days old neonatal pups was dissected under dissecting microscope (Leica GZ6, Germany). Tissues from a minimum of 6 animals were pooled, minced with scissors and incubated in PBS containing 0.25% trypsin and 0.5 mM EDTA, at 37°C for 25 min. The suspension was filtered through 100 μm nylon mesh, washed with cold PBS and plated at a density of 2 × 10^7^ cells in DMEM, 10% FBS, 1% penicillin/streptomycin and 1% L-glutamine. 5 days later microglial cells were shaken off the primary mixed glial cell cultures (250 rpm for 3 hrs at 37°C). Cells were collected by centrifugation, re-suspended in PBS containing 0.2% FBS and eGFP+ microglia were enriched by flow cytometry (BD FACSAria, The University of Chicago Flow Cytometry Facility). Purified microglial cells were plated in culture media supplemented with 5 ng/ml recombinant mouse macrophage-colony stimulating factor (PeproTech, Rocky Hill, NJ) and used for subsequent studies. 3 × 10^5^ microglial cells/well in a 6 well dish were treated with 10 ng/ml of IL-4 (PeproTech, Rocky Hill, NJ) for 24 hrs, later washed and CM was collected in SFM for 24 hrs after initial stimulation. Effect of CM on AHNPC proliferation was determined by BrdU uptake assay as described earlier [12].

## Competing interests

The authors declare no conflict of interest. The corresponding author (S.S.S) discloses that he is a paid Consultant of Eisai Research Labs Inc, but is not a shareholder in any company that is a maker or owner of a FDA-regulated drug or device.

## Authors’ contributions

KV, SHC and SSS designed, performed, analyzed and wrote the paper. XZ assisted in maintaining animal colonies used in this study. All authors read and approved the final manuscript.
